# Pilar Cyst of the Dorsal Hand: A Rare Presentation of a Common Cyst

**DOI:** 10.7759/cureus.60865

**Published:** 2024-05-22

**Authors:** Ryan Ladd, Sarah E Smith, David J DiCaudo, Ramin Fathi, Shari Ochoa

**Affiliations:** 1 Dermatology, Mayo Clinic Arizona, Scottsdale, USA

**Keywords:** isthmus–catagen cyst, atypical location, hand, wens, trichilemmal cyst, pilar cyst

## Abstract

Pilar cysts are common benign cysts of follicular origin that typically arise in areas of skin containing dense hair follicles such as the scalp. Here we describe a unique case of a young woman who was found to have a pilar cyst on the dorsum of her hand, a rather atypical location given the relative lack of pilosebaceous units. This case illustrates the variability in pilar cyst presentation and the importance of considering a pilar cyst in the differential diagnosis of a patient presenting with a tumor of the dorsal hand.

## Introduction

Pilar cysts, also known as trichilemmal cysts, are primarily found on the scalp. They were originally termed “trichilemmal cysts” after Pinkus discovered that these entities originate from the outer root sheath of the hair shaft rather than being of sebaceous origin [1]. An early review of these lesions found that 90% were located on the scalp, with the majority occurring in women. The only lesions to occur elsewhere were located on the forehead or back [2], with subsequent studies reporting similar findings, however, with the remaining ~10% occurring primarily on the posterior neck [3]. Within the literature, there are sparse reports of pilar cysts occurring in sites other than the scalp. Here we present a case of a pilar cyst on the dorsum of the hand, which was clinically concerning for dermatofibrosarcoma protuberans (DFSP). The differential diagnosis also included other benign lesions such as an epidermoid cyst and lipoma. DFSP is a locally aggressive, dermal-based cutaneous soft tissue sarcoma, which can present similarly to a pilar cyst as an asymptomatic, mobile nodule with an overlying violaceous or reddish-brown appearance [4]. DFSP is commonly seen in young adults and demonstrates a similar indolent growth pattern over years, much like pilar cysts. Likewise, case reports have been published describing DFSP masquerading as common cutaneous cysts [5,6]. Because of its similar appearance to more benign skin lesions (dermatofibroma, pilar cyst, epidermal inclusion cyst, etc.), DFSP can be misdiagnosed and unfortunately lead to inadequate treatment.

Of note, this case was previously presented via recorded video abstract at the American Society for Dermatologic Surgery Annual Meeting on October 7, 2022.

## Case presentation

**Figure 1:**
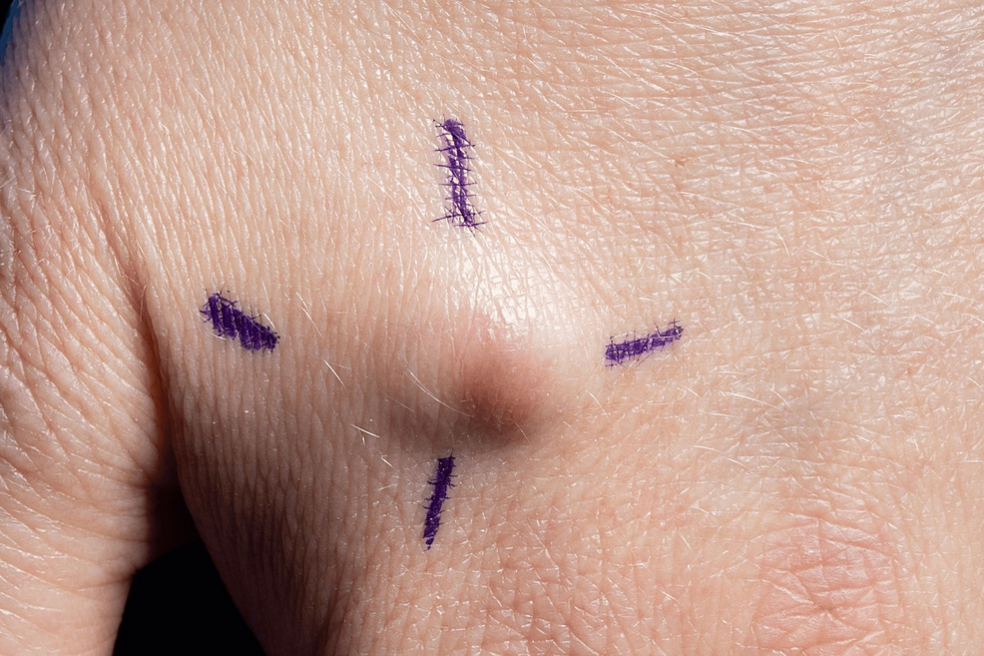
A one-centimeter mobile subcutaneous nodule without overlying puncta.

**Figure 2:**
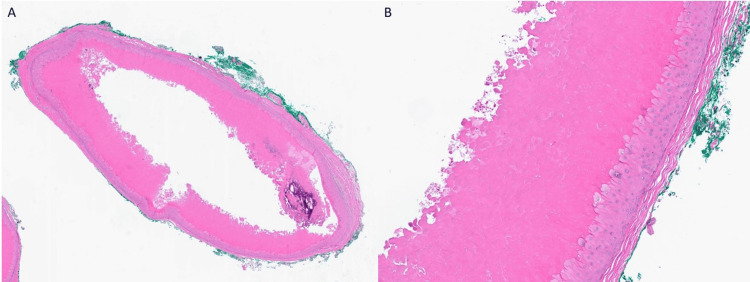
Pilar cyst with focal calcification (A) and trichilemmal keratinization (B), H&E: original magnification x 25 (A), x 200 (B).

A 25-year-old female with a history of rheumatoid arthritis and hypothyroidism presented to the dermatology clinic for evaluation of a lesion on the dorsum of her left hand. Her primary care provider had seen her in the clinic 10 months prior and opted for close monitoring of the lesion; however, given an increase in size in recent months, a referral to dermatology was placed. On presentation, the patient endorsed intermittent episodes of clear drainage from the lesion and tenderness when struck incidentally. She denied preceding trauma or other systemic symptoms. Similarly, she denied a family history of similar-appearing lesions. Examination revealed a one-centimeter mobile, non-tender subcutaneous nodule without an overlying punctum (Figure 1). Differential diagnoses included a pilar cyst, epidermoid cyst, lipoma, and DFSP. Due to concerns of a possible soft tissue malignancy given the atypical location for a pilar cyst, somewhat multinodular appearance of an indurated subcutaneous nodule, slow growth, mild erythema, and risks associated with misdiagnosis, an excisional biopsy was performed under local anesthetic. Pathology was notable for a cystic structure with trichilemmal keratinization, no granular layer, and focal calcification (Figure 2), consistent with a pilar cyst. After excision, the patient had complete resolution and did not require further treatment or follow-up.

## Discussion

Pilar cysts are common, occurring in 5-10% of the population, and account for approximately 20% of all cutaneous cysts [7]. When compared to the more common epidermoid cyst, these lesions are typically firmer and lack a central punctum, although histologic evaluation is ultimately required to make a definitive diagnosis. Clinically, pilar cysts present as well-circumscribed, occasionally lobulated, smooth, round subcutaneous nodules. They may result in overlying alopecia when present on the scalp and can occasionally become inflamed. Pilar cysts have classically been thought to only occur on hair-bearing areas given their trichilemmal origin, with 90% reported to develop on the scalp [3]. Two cases have been reported on the palmar aspect of the fingertips [8,9]. There are also reports of penile, perianal, and eyelid pilar cysts that were preceded by known trauma, raising the question of whether trauma may be an inciting factor in non-traditional areas of the scalp [10-13]. In this patient, the dorsal hand could have been the site of previous trauma; however, she did not report any preceding injury to the area. In general, pilar cysts are known to be benign. However, proliferating trichilemmal cysts, a separate entity from the more typical pilar cyst, can result in aggressive local growth and even malignant transformation in rare instances [14]. For this reason, it is important to identify these lesions. Most cases are sporadic although there are familial pilar cysts that are transmitted in an autosomal dominant pattern with incomplete penetrance. These patients are often found to have a variant of the PLCD1 gene [15]. In cases of familial pilar cysts, patients tend to be younger and multiple lesions are generally present. 

As in this case, histologic evaluation of pilar cysts demonstrates characteristic trichilemmal keratinization: the abrupt maturation of nucleated epithelium to anucleate keratinization with the notable absence of a granular layer [14]. The space within the cyst is composed of homogeneous eosinophilic material. If the capsule ruptures, a granulomatous reaction surrounding the cyst is often seen. The mainstay of treatment is complete surgical excision when the cyst is noninflamed, ensuring complete removal of the cyst wall to prevent recurrence, followed by pathologic evaluation to confirm the diagnosis. Pilar cysts are often easier to extract and are less likely to rupture when compared to the more common epidermoid cysts, which can further guide the diagnosis at the time of excision.

## Conclusions

Overall, this case highlights the clinical challenge and rare presentation of a pilar cyst involving the dorsal hand, an area of skin that lacks dense hair follicles, which are typically implicated in the development of these cutaneous cysts. For this reason, clinicians should avoid excluding the possibility of a pilar cyst solely based on anatomic site and must consider the diagnosis in a patient presenting with a tumor of the dorsal hand.
